# Comparison of the metabolism of 10 chemicals in human and pig skin explants

**DOI:** 10.1002/jat.3730

**Published:** 2018-10-21

**Authors:** C. Géniès, E. L. Jamin, L. Debrauwer, D. Zalko, E. N. Person, J. Eilstein, S. Grégoire, A. Schepky, D. Lange, C. Ellison, A. Roe, S. Salhi, R. Cubberley, N. J. Hewitt, H. Rothe, M. Klaric, H. Duplan, C. Jacques‐Jamin

**Affiliations:** ^1^ Pierre Fabre Dermo‐Cosmétique Toulouse France; ^2^ Coty Darmstadt Germany; ^3^ Toxalim (Research Centre in Food Toxicology) Université de Toulouse, INRA, ENVT, INP‐Purpan, UPS Toulouse France; ^4^ L’Oreal, Aulnay‐Sous‐Bois France; ^5^ Beiersdorf AG Hamburg Germany; ^6^ The Procter & Gamble Company Cincinnati OH USA; ^7^ GSK Nyon Switzerland; ^8^ Unilever Sharnbrook UK; ^9^ Cosmetics Europe Brussels Belgium

**Keywords:** comparison, cosmetics chemicals, human, pig, skin metabolism

## Abstract

Skin metabolism is important to consider when assessing local toxicity and/or penetration of chemicals and their metabolites. If human skin supply is limited, pig skin can be used as an alternative. To identify any species differences, we have investigated the metabolism of 10 chemicals in a pig and human skin explant model. Phase I metabolic pathways in skin from both species included those known to occur via cytochrome P450s, esterases, alcohol dehydrogenases and aldehyde dehydrogenases. Common Phase II pathways were glucuronidation and sulfation but other conjugation pathways were also identified. Chemicals not metabolized by pig skin (caffeine, IQ and 4‐chloroaniline) were also not metabolized by human skin. Six chemicals metabolized by pig skin were metabolized to a similar extent (percentage parent remaining) by human skin. Human skin metabolites were also detected in pig skin incubations, except for one unidentified minor vanillin metabolite. Three cinnamyl alcohol metabolites were unique to pig skin but represented minor metabolites. There were notable species differences in the relative amounts of common metabolites. The difference in the abundance of the sulfate conjugates of resorcinol and 4‐amino‐3‐nitrophenol was in accordance with the known lack of aryl sulfotransferase activity in pigs. In conclusion, while qualitative comparisons of metabolic profiles were consistent between pig and human skin, there were some quantitative differences in the percentage of metabolites formed. This preliminary assessment suggests that pig skin is metabolically competent and could be a useful tool for evaluating potential first‐pass metabolism before testing in human‐derived tissues.

## Abstract 2

We have investigated the metabolism of 10 chemicals in viable pig and human skin. Phase I and II metabolic pathways were present in skin from both species. Chemicals not metabolized by pig skin were also not metabolized by human skin. Six chemicals metabolized by pig skin were also metabolized to a similar extent by human skin. Pig and human skin produced common metabolites, although some species differences were observed and as their relative amounts differed.

**Abbreviations**
2‐AAF: 2‐acetyl aminofluorene
4‐A‐3‐NP: 4‐amino‐3‐nitrophenol
7‐EC: 7‐ethoxycoumarin
ADH: alcohol dehydrogenase
ALDH: aldehyde dehydrogenase
BSA: bovine serum albumin
CYP: cytochrome P450
DPRA: direct peptide reactivity assay
HRMS: high‐resolution mass spectrometry
IQ: 2‐amino‐3‐methylimidazo[4,5‐f]quinoline
NATs: *N*‐acetyltransferases
SCCS: Scientific Committee on Consumer Safety
SULT: sulfotransferase
UGT: UDPGA‐dependent glucuronosyl transferases
XMEs: xenobiotic metabolizing enzymes.

## 1 INTRODUCTION

The main exposure route of cosmetics ingredients is via the skin and consequently, an estimation of the penetration of a chemical into and through skin (whether this is a worst‐case assumption of 100% as a starting point, in silico estimates or an in vitro measurement) is a prerequisite for making a safety assessment of a cosmetics ingredient (SCCS, 2010). The use of non‐viable frozen skin explants for skin penetration studies has been well established and there are several guidelines detailing the methods (OECD, 2004a,b; SCCS, 2010). However, the impact of metabolism in the viable layers of the skin on local toxicity (e.g., skin sensitization or genotoxicity) is less well studied despite its importance in (1) penetration for some chemicals (e.g., for benzo[*a*]pyrene; Jacques, Perdu, Dorio, et al., 2010), and (2) formation of toxic metabolites, which could have an impact on the safety assessment (e.g., for sensitizers; Moss et al., 2016) and detoxification of the parent chemical (Zeller & Pfuhler, 2014). To address this, the Cosmetic Europe Skin Bioavailability and Metabolism Task Force was set up to develop in vitro skin models to improve the prediction of skin bioavailability and thus potential toxicity of topically applied chemicals for future safety assessments.

Ideally, data for human skin are preferred to extrapolate to human scenarios but the use of freshly excised human skin for experimental investigations has associated ethical and practical limitations, particularly in the supply. Alternative models based on non‐human mammalian species and reconstructed human skin models are available; however, the benefit in skin permeation studies using these models are likely limited due to large differences between human and rodent skin structure and metabolism (Boogaard, Denneman, & Van Sittert, 2000) and the questionable performance of reconstructed human skin models as a barrier (Godin & Touitou, 2007). As recommended by the Scientific Committee on Consumer Safety (SCCS, 2010) in case the supply of human skin is limited, pig skin could be a valuable alternative skin model. Pig skin is considered appropriate as it is very close to human skin from histological and physiological viewpoints (Abd et al., 2016; Barbero & Frasch, 2009) and is a waste product of the meat industry (and therefore, ethically sourced in unlimited amounts). Pig skin can be considered similar to human skin in terms of metabolism as both are shown to express phase I enzymes, including cytochrome P450s (CYPs), a key oxidative enzyme family in the metabolism of xenobiotics, and phase II enzymes, including UDP‐glucuronyl transferases (UGT) and sulfotransferases (SULT), the main classes involved in the detoxification (Oesch, Fabian, Guth, & Landsiedel, 2014; Jacques, Perdu, Dorio, et al., 2010; Jacques, Perdu, Duplan, et al., 2010). In vitro pig skin was also shown to be active metabolically towards xenobiotics with widely differing physicochemical properties, namely 7‐ethoxycoumarin, benzo[*a*]pyrene and testosterone (Jacques, Perdu, Dorio, et al., 2010; Jacques, Perdu, Duplan, et al., 2010; Jacques et al., 2014). There are only a few reports comparing the metabolism of chemicals in human and pig skin (Jewell et al., 2007; Oesch et al., 2014; Prusakiewicz, Ackermann, & Voorman, 2006; Rolsted, Kissmeyer, Rist, & Hansen, 2008); therefore, the aim of the current work was to determine if pig skin is a suitable alternative to human skin in assessing the metabolism of topically applied chemicals.

In these studies, we have compared the distribution and metabolism of 10 chemicals in pig and human skin using an in vitro model. The in vitro skin model consists of a skin explant from pig or human donors seeded dermal side down in inserts and placed in six‐well plates. These human and pig skin explants have been shown to express functional phase I and II xenobiotic metabolizing enzyme (XME) activities associated with a good conservation of the skin barrier function (Jacques, Perdu, Dorio, et al., 2010; Jacques, Perdu, Duplan, et al., 2010; Jacques et al., 2014). One advantage of this assay is that it can be used to measure simultaneously the dose‐dependent distribution and metabolism of radiolabeled chemicals applied to the surface of the skin over 24 hours, thus representing the in vivo application scenario and covering slower metabolism pathways. The chemicals tested, which were mostly cosmetics ingredients or reference chemicals for toxicity assays, exhibited a range of physicochemical properties (Table 1). These chemicals were selected because there are a number of XME pathways by which they could be expected to be metabolized, based on the published literature for known metabolism in vivo and/or in hepatic models in vitro (as described and cited in Section 3). The pathways include phase I (e.g., CYPs, alcohol dehydrogenases [ADH], aldehyde dehydrogenases [ALDH], esterases) and phase II conjugation (e.g., sulfation and glucuronidation) reactions. Radiolabeled chemicals were used to quantify the disappearance of parent compounds and metabolites formed, which were identified by high‐resolution mass spectrometry (HRMS).

**Table 1:** Use, some physicochemical properties and doses of the chemicals tested in in vitro skin experiments

Chemical (CAS number)	Use	Expected metabolic pathway XMEs	MW	LogP	Maximum water solubility (mg/mL)	Solvent	Dose (nmol/cm^2^)	
							Pig	Human
Caffeine (58‐08‐2)	Cosmetic	CYPs	194.2	−0.07	17.5	0.01 M PBS	2.4	5.0
4‐Chloroaniline (106‐47‐8)	Reference chemical, skin sensitizer and genotoxic	CYPs	127.6	1.83	3.2	0.1 M PBS	2.4	3.0
2‐Amino‐3‐methylimidazo[4,5‐f] quinoline (76180‐96‐6)	Reference chemical, genotoxic	CYPs	198.2	1.47	0.53	0.01 M PBS	2.4	2.4
7‐Ethoxycoumarin (31005‐02‐4)	Reference chemical, non‐toxic	CYPs, UGT, SULT	190.2	2.30	0.78	0.01 M PBS	2.4	2.5
2‐Acetyl aminofluorene (53‐96‐3)	Reference chemical, genotoxic	CYPs, SULT	223.3	3.10	0.004 (9.6 in ethanol)	100% Ethanol	2.4	2.6
Resorcinol (108‐46‐3)	Cosmetic	UGT, SULT	110.1	0.80	504	0.1 M PBS	2.4	8.7
4‐Amino‐3‐nitrophenol (610‐81‐1)	Cosmetic	UGT, SULT	154.1	0.41	2.0	0.01 M PBS	2.4	2.5
Cinnamyl alcohol (104‐54‐1)	Cosmetic	ADH, ALDH, CYPs	134.2	1.95	4.0	0.01 M PBS	2.4	12.2
Propyl paraben (94‐13‐3)	Cosmetic	Esterase,UGT, SULT	180.2	3.04	0.52	0.01 M PBS	2.4	2.7
Vanillin (121‐33‐5)	Cosmetic	AO, ADH, UGT, SULT	152.2	1.21	8.5	0.01 M PBS	2.4	6.3

ADH, alcohol dehydrogenase; ALDH, aldehyde dehydrogenase; AO, aldehyde oxidase; CYPs, cytochrome P450s; PBS, phosphate‐buffered saline; SULT, sulfotransferases; UGT, UDP‐glucuronyl transferases; XMEs, xenobiotic metabolizing enzymes.

Incubations with pig skin were conducted before those with human skin and, therefore, the doses used in pig skin incubations were set to 2.4 nmol/cm^2^. Doses used for human skin were based on the dose tested in skin penetration studies. Physicochemical properties were from Chemspider (www.chemspider.com).

## 2 MATERIALS AND METHODS

### 2.1 Chemicals

Ring‐labeled ^14^C radiolabeled chemicals were from either of the following suppliers. American Radiolabeled Chemicals, Inc. (St. Louis, MO, USA): caffeine[8‐^14^C] (55 mCi/mmol, purity 99%); resorcinol[^14^C(U)] (55 mCi/mmol, purity 98%); 7‐ethoxycoumarin (7‐EC) phenyl ring‐^14^C(U)] (55 mCi/mmol, purity 99%); 4‐chloroaniline[^14^C(U)]HCl (25 mCi/mmol, purity 99%); and vanillin [carbonyl‐^14^C] (56 mCi/mmol, purity 98.0%). Selcia (Ongar, Essex, UK): ([9‐^14^C]‐2‐acetylaminofluorene (58.58 mCi/mmol, purity 92.7%); trans‐[phenyl‐U‐^14^C] cinnamyl alcohol (30.41 mCi/mmol, purity 99.9%); 4‐amino‐3‐nitro[^14^C(U)]phenol (27.73 mCi/mmol, purity 99.4%); [2‐^14^C]‐2‐amino‐3‐methyl‐3H‐imidazo[4,5‐F] quinoline (IQ) (46.6 mCi/mmol, purity 99.7%). Quotient Bioresearch Ltd. (Cardiff, UK): propyl[^14^C] paraben (51 mCi/mmol, purity 99.3%). Label‐free chemicals were all from Sigma‐Aldrich (St. Louis, MO, USA); with the exception of IQ, which was from Chemos GmbH (Regenstauf, Germany). All other chemicals and solutions used were from Sigma‐Aldrich. Radiolabeled chemicals, as tracers, were mixed with label‐free chemicals to achieve the final concentrations.

### 2.2 Skin tissue

Human abdominal skin was obtained (with consent for research from a commercial supplier [L’Union Clinic, Rangueil hospital and Icelltis, Toulouse, France] with certified ethical procedures) from six male and 34 female healthy donors, 41 ± 11 years of age, undergoing cosmetic surgery. Abdominal skin was kept at 4°C during transports and placed in culture within 3 hours of surgery. Pig ear skin was a waste product of the meat industry (without hot steam treatment) from domestic pigs (female Pietrain breed, 6 months old, 80‐100 kg) obtained from a local slaughterhouse (Montauban, France). Ears were taken from the animals within 5 minutes of slaughter and were kept at 4°C during transport to the laboratory, which lasted a maximum of 2 hours. Pig skin samples were dried and then gently shaved.

### 2.3 Skin incubations

The experiment and the preparation of the culture medium were performed under sterile conditions. Human and pig skin was cut with a dermatome to a thickness of 450 ± 50 μm. Skin punches were seeded dermal side down in polycarbonate Transwell^®^ inserts from Corning Life Sciences (Avon, France; 4.16 cm^2^ application area with an 8 μm pore size filter). Preliminary tests showed that the inclusion of a plastic border of the well ensures no leakage of the dose solution into the receiver compartment occurs. The inserts were placed in a six‐well plate containing 1.5 mL culture medium and the skin explants incubated in a standard incubator at 37°C, under an atmosphere of 5% CO_2_ and 50 ± 10% relative humidity. The culture medium was Dulbecco's modified Eagle medium without phenol red supplemented with 8 mm l‐glutamine, 2 μg/mL streptomycin/penicillin, 25 ng/mL fungizone and 50 ng/mL gentamycin. Skin explants were incubated for 1 hour before application of the formulation. Before the main study, the sink conditions were checked, i.e. confirmation that the concentrations in the medium resulting from the highest doses of chemicals were 10‐fold lower than their maximum solubility in culture medium were checked before the beginning of the study. This was to ensure they did not influence the delivery of the test compound. Based on these data, bovine serum albumin (BSA), 4% (w/v) was included in the medium for some chemicals (4‐chloroaniline, IQ and 2‐acetyl aminofluorene [2‐AAF]) to increase their solubility in the culture medium.

The doses tested in pig and human skin are listed in Table 1. The dosing volume was 10 μL/cm^2^. For pig skin metabolism studies, which were carried out before the human skin experiments to ensure the analytics were optimal, 2.4 nmol/cm^2^ of each chemical (only the ^14^C‐labeled form) was applied on the skin and incubated for 24 hours for the measurement of metabolites in the medium. These tissues were further incubated for a total of 48 hours, with a replacement of the culture medium after 24 hours. This longer incubation time and the standard doses of 2.4 nmol/cm^2^ were used to maximize the likelihood of the skin forming metabolites to develop analytical methods. The metabolites profiles in pig and human skin were compared in the medium sample at 24 hours of incubation. The metabolite profiles for the 48‐hour timepoint are not reported here since humans skin metabolism was measured at 24 hours only; however, for completeness, the distribution of radioactivity at the end of both pig (48 hours) and human (24 hours) skin incubations is reported. The doses (and total incubation duration of 24 hours) for human skin were based on solubility studies and included those used in skin penetration studies using frozen human skin, which were run in parallel (for which higher amounts of radioactivity were required, resulting in higher doses used for human skin). The doses were obtained by mixing radiolabeled and cold chemical. One replicate disc per donor and per dose was used. All chemicals were tested on four donors of pig and human skin, except for 2‐AAF and 4‐amino‐3‐nitrophenol (4‐A‐3‐NP), for which three donors were used. The medium was removed after 24 hours of incubation and stored at −20°C for analysis.

### 2.4 Skin histology and integrity

It has been shown that the integrity of skin explants using MTT and lactate dehydrogenase can be maintained for up to 72 hours (Jacques, Perdu, Dorio, et al., 2010; Jacques, Perdu, Duplan, et al., 2010). Likewise, we confirmed that the integrity of non‐treated skin explants were maintenance of skin integrity over 24 hours (human) and 48 hours (pig) using histological analysis. Skin samples were fixed in Bouin's liquid, embedded in paraffin, cut into 5 μm sections and stained with hematoxylin and eosin (Rijnkels, Whiteley, & Beijersbergen van Henegouwen, 2001). Morphological examinations were carried out using a Nikon eclipse E600 microscope equipped with a Dxm1200 digital camera. Images were collected at 50× magnification using Lucia G version 4.82 software.

### 2.5 Metabolite analysis

Medium samples (50 μL) from the medium samples taken after 24 hours incubation were extracted using an equal volume of 100% acetonitrile (caffeine, resorcinol, 7‐EC, propyl paraben, 2‐AAF and cinnamyl alcohol); 90% 0.1 m phosphate‐buffered saline (PBS) + 10% acetonitrile/acetone (1:3) (4‐chloroaniline); 80% 0.1 m PBS + 20% acetonitrile (4‐A‐3‐NP); 90% 0.1 m PBS + 10% acetonitrile (IQ) or 100% ethanol (vanillin). After centrifugation, a 100 μL aliquot of the extract was counted by liquid scintillation. The pellet was dissolved in 3 mL Soluable (60°C, 24 hours) and then counted by liquid scintillation.

Analysis of the chemicals and their metabolites in medium after 24 hours of incubation with pig and human skin was measured by radio‐high‐performance liquid chromatography (HPLC) using an HPLC 1100 Series, Agilent and a Flow Scintillation Analyzer, Radiomatic 610 TR, Perkin Elmer (Villebon‐sur‐Yvette, France). There were slight differences in the retention times of peaks between pig and human samples due to a new column being used for the human samples, which was most noticeable for propyl paraben. To confirm that metabolites were the same in pig and human skin, medium samples were co‐injected on the new column. The metabolites were identified by LC‐electrospray ionization‐HRMS (HPLC‐ESI‐HRMS) with a RSLC3000 HPLC system coupled to a LTQ‐Orbitrap XL mass spectrometer (Thermo Scientific). The HPLC analytical conditions for identification and quantification of the metabolites for each chemical are summarized in Supporting information Table S1. Metabolites were then identified based on their accurate mass measured with an *m/z* error of 5 ppm and based on characteristic fragmentation patterns observed during MS/MS experiments achieved with the collision‐induced dissociation mode of the LTQ‐Orbitrap mass spectrometer.

### 2.6 Cutaneous distribution

At the end of the experiment, the trans‐wells were dismantled and washed with appropriate solvent for each chemical. The skin surfaces were washed three times with half cotton‐swabs soaked with appropriate solvent (50:50 water/ethanol for caffeine and resorcinol, 100% acetonitrile for 7‐EC and 100% ethanol for all other chemicals). The cotton swabs were placed in 5 mL of appropriate solvent in a scintillation counting flask and placed in a sonicator bath for 20 minutes. Aliquots of 1 mL were counted by liquid scintillation, after addition of 15 mL of scintillation liquid. Tissue culture inserts were washed with 10 mL of appropriate solvent. One aliquot of 1 mL was counted by liquid scintillation, after addition of 15 mL of scintillation liquid. After media removal, wells were washed twice with 1 mL of the appropriate solvent. Washing solutions were counted by liquid scintillation, after addition of 15 mL of scintillation liquid.

Pig skin explants were digested using 3 mL Soluable (60°C for 24 hours) and counted by liquid scintillation. Skin extraction process was adapted according to the physicochemical properties of each chemical and its potential metabolites. Therefore, the extraction method for six chemicals tested in human skin involved cryogrinding the skin and then extracting ^14^C‐analytes in 100% acetonitrile. For 4‐chloroaniline, the extraction method included cryogrinding, stirring and ultrasonication, followed by extraction with 0.1 m PBS with 10% acetonitrile/acetone (1:3). 4‐Amino‐3‐nitrophenol ^14^C‐analytes were extracted from human skin by first cryogrinding the skin and then extracting in 0.1 m PBS with 20% acetonitrile. The extraction of IQ ^14^C‐analytes included cryogrinding, stirring and ultrasonication, followed by extraction with 0.1 m PBS with 10% acetonitrile. Vanillin analytes were extracted by cutting the skin into small pieces and then stirring overnight, followed by extraction in 100% ethanol. After centrifugation, an aliquot of 100 μL of the extract was counted by liquid scintillation and the pellet was counted after digestion with 3 mL Soluable (60°C for 24 hours). The amount of culture media was measured exactly with a pipette or by weighing. A volume of 100 μL of medium was counted for radioactivity after the addition of 15 mL scintillation liquid. Radioactivity was determined by direct counting of aliquots on a Packard scintillation counter (Model Tricarb A2900TR; Packard Instruments, Meriden, CT, USA) using Packard Ultima Gold as the scintillation cocktail (Packard Instruments, Downer Grove, IL, USA). For all vials, sample quenching was compensated by the use of quench curves and external standardization. Media were stored at −20°C until radio‐HPLC analysis.

### 2.7 Statistical analysis

The data were analyzed using Graphpad Prism 7 (Graphpad Software, San Diego, CA, USA). Results were expressed as mean ± SEM. For comparison between pig (*n* = 3 or 4) and human skin (*n* = 4), data were analyzed using the non‐parametric Mann‐Whitney *U*‐test. *P* < 0.05 was regarded as statistically significant.

## 3 RESULTS

### 3.1 Cutaneous distribution of chemicals

The majority of the applied doses of chemicals were recovered in the medium 24 hours after application to pig and human skin (Table 2). No statistical difference was observed in the percentage of radioactivity in the medium between human and pig skin 24 hours after application except for cinnamyl alcohol, which was likely due to a difference in the applied dose (Table 1). The distribution of each chemical in the skin wash, skin and medium 48 hours after application to pig and human skin is summarized in Supporting information Table S2. Although the total amount of radioactivity in the skin and medium were comparable in pig (48 hours) and human skin incubation (24 hours), there was a relatively higher amount in the medium than in the skin compartment in pig skin incubations compared to human skin incubations. For example, in pig skin incubations, the percentage of the applied dose of 4‐chloroaniline in the medium and skin was 82% and 16%, respectively, whereas, in human skin, the percentage of the applied dose of 4‐chloroaniline in the medium and skin was 50% and 35%, respectively. This was likely due to the longer incubation for pig skin, which allowed for further absorption of the radioactivity into the medium below the skin. This finding was also observed for all the chemicals tested (Supporting information Table S2).

**Table 2:** Mass balance and amount of the radioactivity present in the skin and medium after application to pig and human skin

Chemical	Mass balance (% of applied dose at end of incubation)		% Radioactivity in culture media after 24 h	
	Pig (48 h)	Human (24 h)	Pig	Human
Caffeine	109.2 ± 3.3	104.0 ± 0.7	72.0 ± 4.0	81.4 ± 3.3
4‐Chloroaniline	99.2 ± 10.8	86.2 ± 0.7	54.3 ± 0.6	49.7 ± 4.4
IQ	96.3 ± 6.6	94.5 ± 10.6	30.1 ± 2.3	10.5 ± 13.4
7‐EC	100.0 ± 3.8	91.9 ± 3.8	62.5 ± 2.7	66.0 ± 0.9
2‐AAF	87.8 ± 1.1	91.6 ± 4.5	52.8 ± 2.3	48.3 ± 4.2
Resorcinol	92.7 ± 5.92	94.2 ± 3.5	58.6 ± 7.6	54.4. ± 1.5
4‐Amino‐3‐nitrophenol	96.0 ± 2.6	94.4 ± 3.0	51.9 ± 6.7	62.3 ± 3.4
Cinnamyl alcohol	96.8 ± 1.9	99.9 ± 5.8	63.2 ± 1.9	83.5 ± 4.8*
Propyl paraben	98.7 ± 3.3	91.8 ± 6.6	53.3 ± 2.5	66.0 ± 9.2
Vanillin	90.7 ± 1.7	104.4 ± 6.0	58.6 ± 5.2	60.0 ± 7.8

2‐AAF, 2‐acetyl aminofluorene; 7‐EC, 7‐ethoxycoumarin; IQ, 2‐amino‐3‐methylimidazo[4,5‐f]quinoline.

* Statistical difference between pig and human skin (*P* < 0.05).

### 3.2 Metabolism

### 3.3 Cytochrome P450 substrates

**Figure 1:**
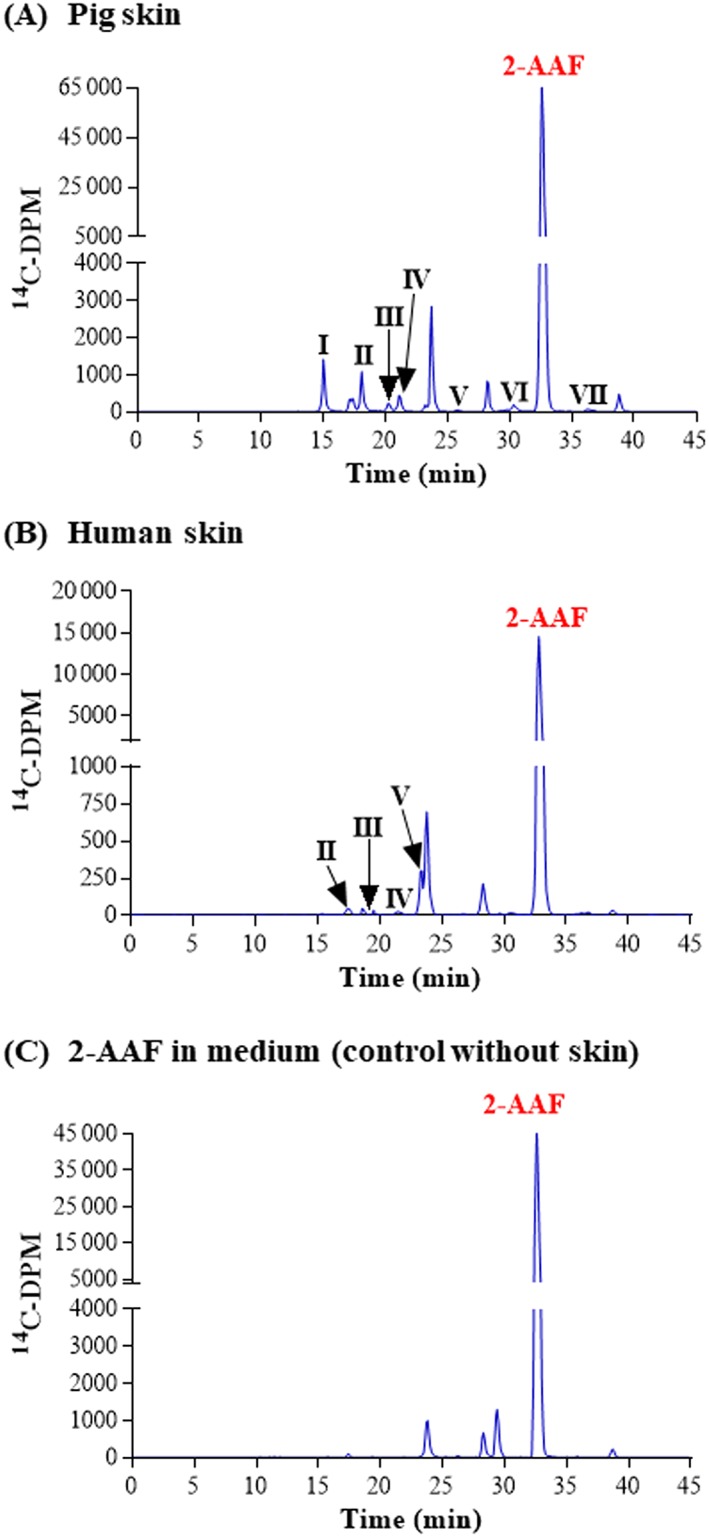
2‐AAF metabolism in pig and human skin. A, Pig skin. B, Human skin. Radiochromatograms of the medium collected at 24 h from pig and human skin incubations. C, Radiochromatogram showing the impurities of radiolabeled 2‐AAF present in medium from 24 h incubations without skin. 2‐AAF, 2‐acetyl aminofluorene [Colour figure can be viewed at wileyonlinelibrary.com]

It is known that caffeine, 4‐chloroaniline and IQ are metabolized by CYPs, in particular CYP1A2. In our in vitro skin model regardless of species, no metabolites were detected in the skin or culture media. 2‐AAF is also metabolized by CYP1A1/2 in human liver microsomes (McManus et al., 1990); however, although we were able to show the presence of metabolites in the medium of incubations with pig and human skin (Figure 1A and 1B), we could not confirm that these were a result of 2‐AAF metabolism. The reason for this was the presence of some impurities in the control culture medium in the absence of skin explants (Figure 1C). The purity of the radiolabeled form of this chemical was 92.7%. Therefore, the molecules detected could have been metabolites of the impurities rather than of 2‐AAF itself (for this reason, the amounts of each are not shown).

### 3.4 Cytochrome P450 and sulfotransferases/UDP‐glucuronyl transferases substrate

**Figure 2:**
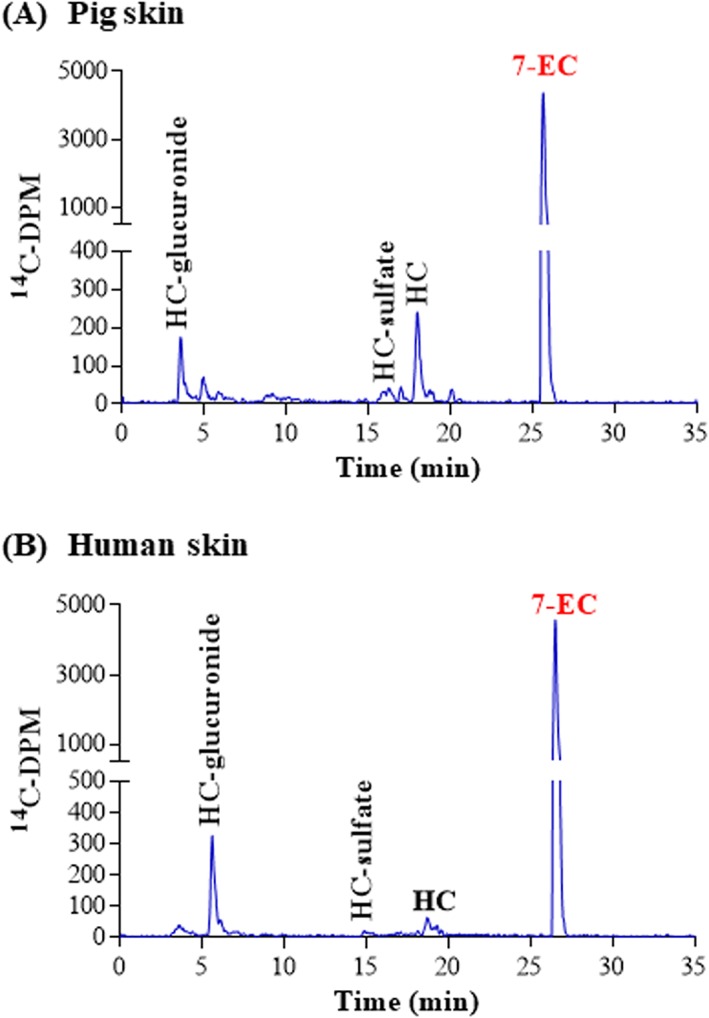
7‐EC metabolism in pig and human skin. A, Pig skin. B, Human skin. Radiochromatograms of the medium collected at 24 h from pig and human skin incubations. 7‐EC, 7‐ethoxycoumarin; HC, hydroxycoumarin [Colour figure can be viewed at wileyonlinelibrary.com]

The metabolism of 7‐EC in the liver is reported via several CYPs (mainly CYP1A1 and CYP2E1 and a minor amount via CYP1A2 and CYP1B1) to 7‐hydroxycoumarin (HC) (Shimada et al., 1997), followed by conjugation of HC via SULT and UGTs (De Kanter et al., 1999). This pathway was also demonstrated with our in vitro skin model for pig and human with 7‐EC. Although a large percentage of the radioactivity present in the medium was the parent chemical (~60% of the applied dose; Table 3), 7‐EC was metabolized to three classes of metabolites (representing less than 10% of parent applied dose; Figure 2). The metabolites were identified as the phase I‐mediated metabolite, 7‐HC and two phase II metabolites: the glucuronide and sulfate conjugate of HC. The relative amounts of hydroxylated metabolites in pig and human skin incubations were equivalent but a statistical difference was observed for the conjugated metabolites (*P* > 0.05) (Table 3).

**Table 3:** Amount of 7‐EC and its metabolites in the medium after 24 h

	% Applied dose	
	Pig	Human
Parent compound + metabolites	69.8 ± 0.1	62.2 ± 0.3
7‐EC (parent compound)	60.8 ± 0.6	57.7 ± 3.1
Total metabolites	9.0 ± 0.6	4.5 ± 1.3
HC‐glucuronide	4.9 ± 0.9	2.2 ± 1.2*
HC‐sulfate	2.6 ± 0.9	0.9 ± 0.6*
HC	1.5 ± 0.8	1.4 ± 0.4

7‐EC, 7‐ethoxycoumarin; HC, hydroxycoumarin.

Amounts of metabolites in the medium expressed as a percentage of the applied dose, mean ± SEM, four donors, *n* = 1 per donor.

* Statistical difference between pig and human skin (*P* < 0.05).

### 3.5 Sulfotransferase/UDP‐glucuronyl transferase substrates

**Figure 3:**
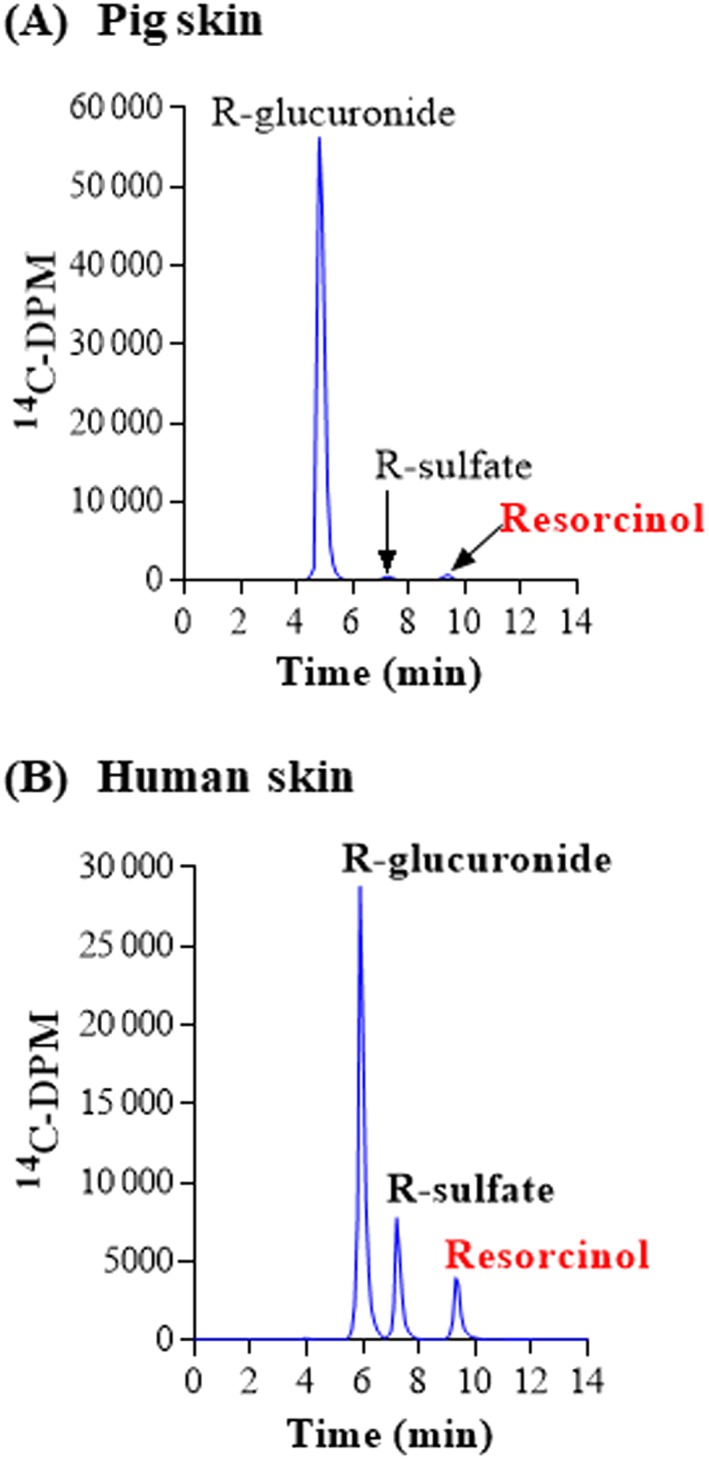
Resorcinol metabolism in pig and human skin. A, Pig skin. B, Human skin. Radiochromatograms of the medium collected at 24 h from pig and human skin incubations. R, resorcinol [Colour figure can be viewed at wileyonlinelibrary.com]

**Figure 4:**
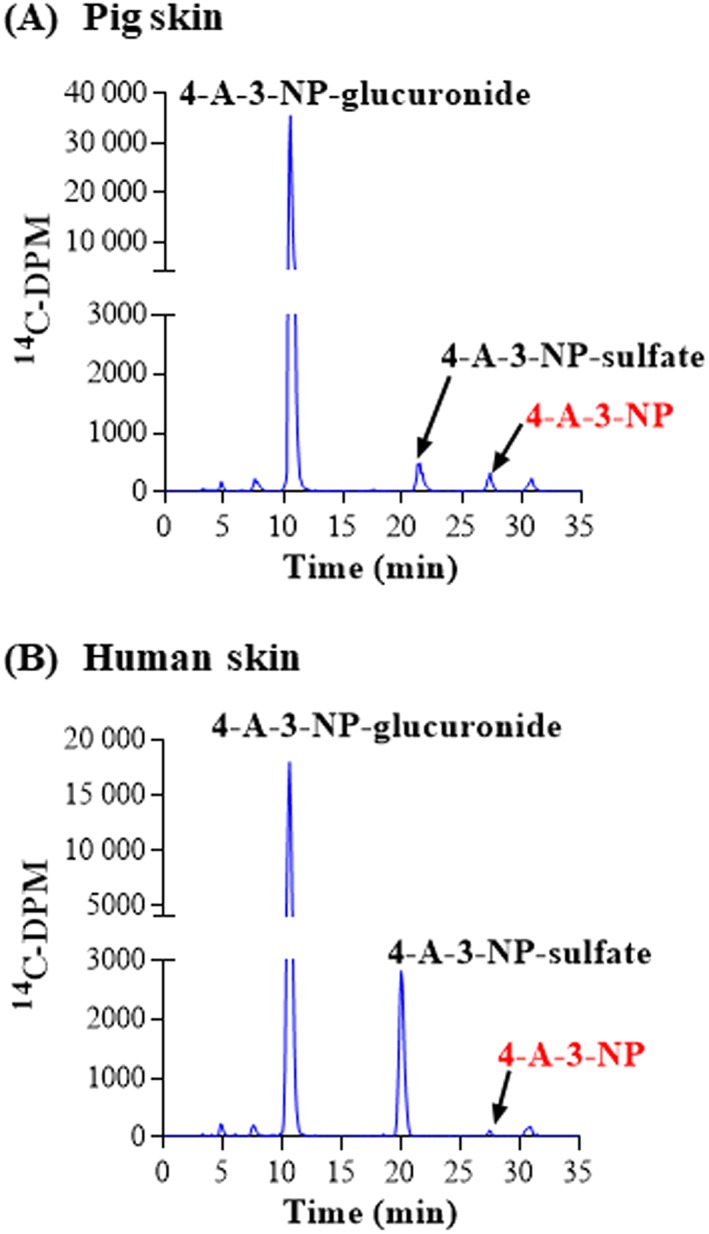
4‐A‐3‐NP metabolism in pig and human skin. A, Pig skin. B, Human skin. Radiochromatograms of the medium collected at 24 h from pig and human skin incubations. 4‐A‐3‐NP, 4‐amino‐3‐nitrophenol [Colour figure can be viewed at wileyonlinelibrary.com]

There were two chemicals that were metabolized to only two conjugated metabolites via UGTs and SULTs, namely resorcinol and 4‐A‐3‐NP. For both chemicals, almost all the radioactivity present in the medium after 24 hours (which was ~40%‐50% of the applied dose; Table 4) was attributed to metabolites, and very little of the parent chemicals remained. The major metabolite of both chemicals formed in skin from both species was the glucuronide conjugate (>78% of the total metabolites observed) and a minor metabolite was the sulfate conjugate (Figures 3 and 4). The contribution of the sulfate pathway to the metabolism was higher in human than in pig skin: for resorcinol, the sulfate represented 19% of the total metabolites compared to only 1% of the total metabolites in pig skin incubations (Table 4; *P* < 0.05) and, for 4‐A‐3‐NP, the sulfate represented 22% of the total metabolites compared to only 4% of the total metabolites in pig skin incubations (Table 5; *P* < 0.05).

**Table 4:** Amount of resorcinol and its metabolites in the medium after 24 h

	% Applied dose	
	Pig	Human
Parent compound + metabolites	50.9 ± 1.2	46.7 ± 0.7
R (parent compound)	1.8 ± 0.8	4.8 ± 0.7
Total metabolites	49.1 ± 0.8	41.9 ± 0.6
R‐glucuronide	48.6 ± 0.8	33.7 ± 0.6*
R‐sulfate	0.5 ± 0.03	8.2 ± 0.1*

R, resorcinol.

Amounts of metabolites in the medium expressed as a percentage of the applied dose, mean ± SEM, four donors, *n* = 1 per donor.

* Statistical difference between pig and human skin (*P* < 0.05).

**Table 5:** Amount of 4‐A‐3‐NP and its metabolites in the medium after 24 h

	% Applied dose	
	Pig	Human
Parent compound + metabolites	47.9 ± 0.1	45.1 ± 0.7
4‐A‐3‐NP (parent compound)	0.6 ± 0.2	0.1 ± 0.06
Total metabolites	47.3 ± 0.2	45.0 ± 0.7
4‐A‐3‐NP‐glucuronide	45.5 ± 0.2	35.0 ± 2.4*
4‐A‐3‐NP‐sulfate	1.8 ± 0.3	9.98 ± 2.7*

4‐A‐3‐NP, 4‐amino‐3‐nitrophenol.

Amounts of metabolites in the medium expressed as a percentage of the applied dose, mean ± SEM, three pig and four human donors, *n* = 1 per donor.

* Statistical difference between pig and human skin (*P* < 0.05).

Interestingly, while several structurally related aromatic amines are readily *N*‐acetylated in in vitro skin models, 4‐A‐3‐NP differs from these and is not a substrate for the *N*‐acetyltransferase (NAT)1 enzyme, possibly due to steric hindrance of the side groups (unpublished data). For this reason, an *N*‐acetylated metabolite of 4‐A‐3‐NP was not expected, and indeed, was not detected in these incubations with pig or human skin.

### 3.6 Esterase and sulfotransferase/UDP‐glucuronyl transferase substrates

**Figure 5:**
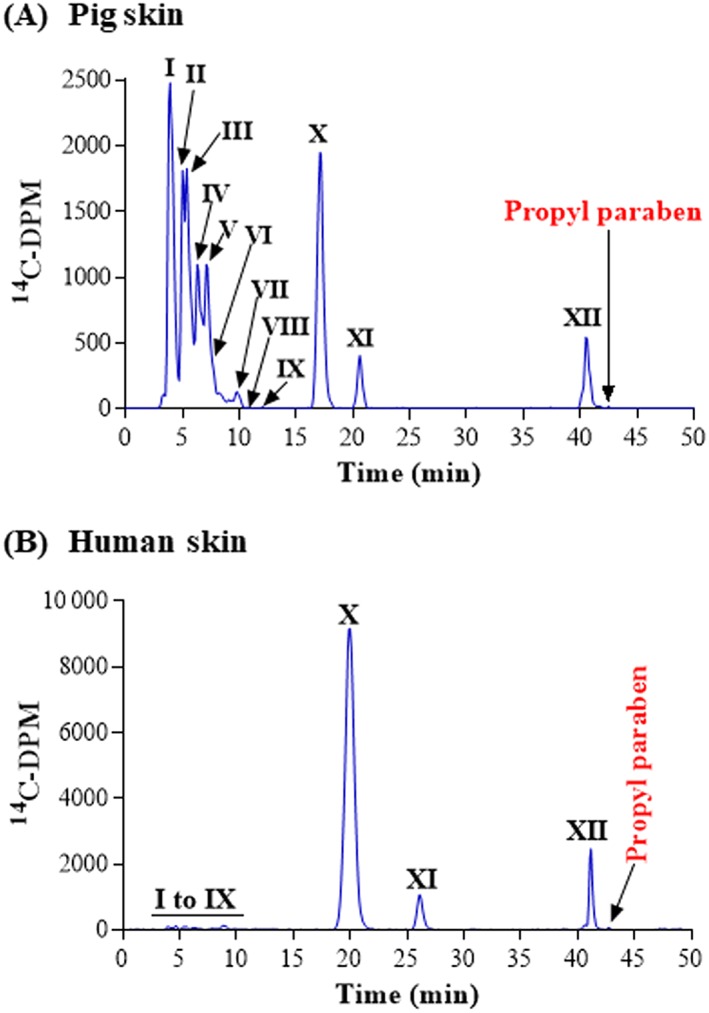
Propyl paraben metabolism in pig and human skin. A, Pig skin. B, Human skin. Radiochromatograms of the medium collected at 24 h from pig and human skin incubations. There was a slight difference in the retention times of the peaks between the samples shown due to a new column being used for the human samples. To confirm that metabolites were the same in pig and human skin, medium samples were co‐injected on the new column. I and VI, 4‐hydroxybenzoic acid glucuronide conjugate; X, 4‐hydroxybenzoic acid; XII, propylparaben sulfate conjugate [Colour figure can be viewed at wileyonlinelibrary.com]

It has been reported that propyl paraben is converted to several metabolites (Abbas et al., 2010; Bando, Mohri, Yamashita, Takakura, & Hashida, 1997; Jewell et al., 2007). It can be directly conjugated to form propyl paraben‐sulfate and glucuronide; or conjugates can be formed after ester cleavage to 4‐hydroxybenzoic acid. In our studies, this chemical passed through pig and human skin, such that ~50% of the applied radioactivity was recovered into the culture medium after 24 hours (Table 6). There was also extensive metabolism and nearly all propyl paraben applied to pig and human skin was subsequently present in the medium as metabolites (Figure 5, Table 6). Twelve metabolites were detected in the culture medium, of which the four major metabolites were identified by HRMS (Figure 5, Table 6). The total percentage of metabolism was equivalent between pig and human skin (*P* > 0.05). The two major metabolites that were identified in pig skin (peak I) were the glucuronide conjugate of 4‐hydroxybenzoic acid (12.8% of the applied dose) and the non‐conjugated 4‐hydroxybenzoic acid (peak X), which represented 9.5% of the applied dose. The two other metabolites identified were 4‐hydoxybenzoic acid glucuronide (peak VI) and propyl paraben sulfate (peak XII), representing 5.0% and 2.7% of the applied dose, respectively. There were metabolites that were not identified that eluted between peak I and peak VI, representing between 1.68% and 34.8% of applied dose. All 12 metabolites that were produced in incubations with pig skin were also produced in human skin; however, the relative proportion of each differed. The same major metabolites were recovered in pig and human skin including 4‐hydroxybenzoic acid (peak X), peak XI and the sulfate conjugate of the propyl paraben (peak XII). The metabolites I‐IX were recovered in human skin in smaller amounts than in pig skin and each represented less than 0.5% of the applied dose (Figure 5, Table 6).

**Table 6:** Amount of propyl paraben and its metabolites in the medium after 24 h

	% Applied dose	
	Pig	Human
Parent compound + metabolites	50.3 ± 1.1	56.0 ± 2.6
Propyl paraben (parent compound)	0.0	0.2 ± 0.2
Total metabolites	50.3 ± 1.1	55.8 ± 2.9
I (HBA‐glucuronides)	12.9 ± 0.3	0.02 ± 0.01*
II (maleic acid)	5.6 ± 0.3	0.5 ± 0.03*
III (not identified)	6.5 ± 0.4	0.3 ± 0.1*
IV (not identified)	3.6 ± 0.1	0.3 ± 0.06*
V (not identified)	1.3 ± 0.2	0.4 ± 0.04
VI (HBA‐glucuronides)	5.0 ± 0.05	0.15 ± 0.03*
VII (not identified)	0.4 ± 0.05	0.3 ± 0.2
VIII (not identified)	0.1 ± 0.01	0.4 ± 0.04*
IX (not identified)	0.5 ± 0.1	0.3 ± 0.1
X (4‐HBA)	9.5 ± 0.6	42.09 ± 2.8*
XI (not identified)	2.3 ± 0.7	5.31 ± 0.9*
XII (propyl paraben sulfate)	2.7 ± 1.4	5.67 ± 0.3*

HBA, hydroxybenzoic acid.

Amounts of metabolites in the medium expressed as a percentage of the applied dose, mean ± SEM, four donors, *n* = 1 per donor.

* Statistical difference between pig and human skin (*P* < 0.05)

### 3.7 Alcohol and aldehyde dehydrogenase activities and/or sulfotransferases/UDP‐glucuronyl transferases

**Figure 6:**
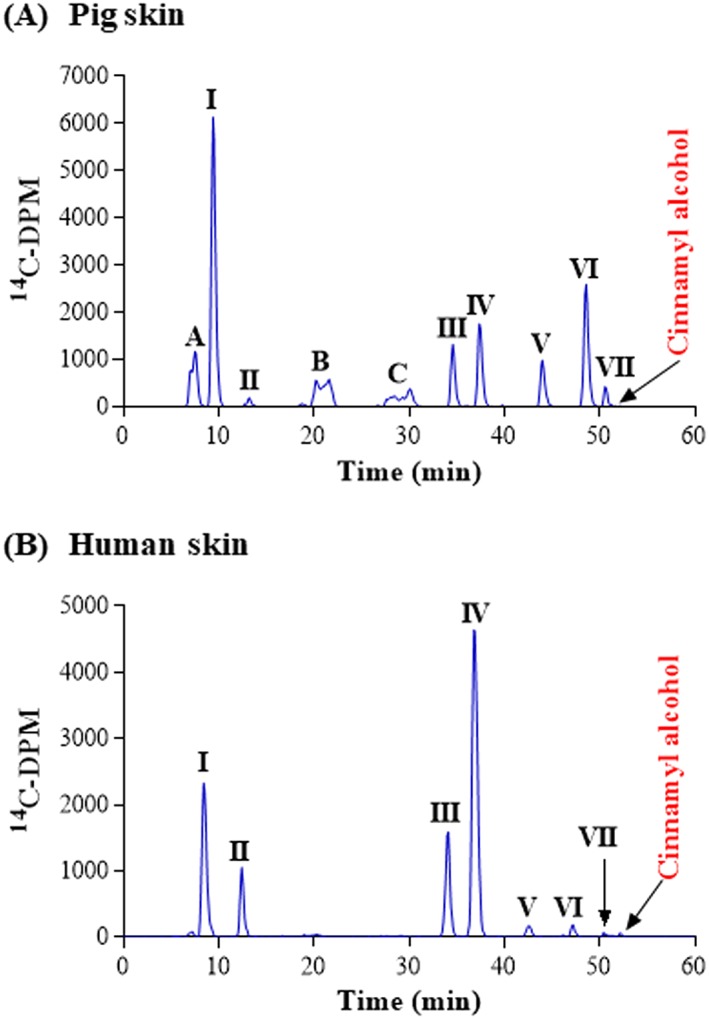
Cinnamyl alcohol metabolism in pig and human skin. A, Pig skin. B, Human skin. Radiochromatograms of the medium collected at 24 h from pig and human skin incubations. B, benzoic acid; I, 3‐hydroxy‐3‐phenylacrylic acid; II, benzoic acid glucuronide; III, OH‐cinnamic acid‐cysteine glucuronide; IV, 3‐hydroxy‐3‐phenylpropanoic acid with OH‐cinnamyl alcohol‐glutathion (or 3‐hydroxypropanoic acid‐glutathion); V, benzoylglycine; VI, cinnamic acid; VII, cinnamic acid glucuronide [Colour figure can be viewed at wileyonlinelibrary.com]

It is known that cinnamyl alcohol is converted rapidly to cinnamaldehyde via ADH, which, then is converted to cinnamic acid by ALDH. Cinnamyl alcohol can also be metabolized to an epoxy‐alcohol and an allylic hydroxy‐metabolite via CYPs (Moss et al., 2016; Niklasson, Ponting, Luthman, & Karlberg, 2014). Cinnamyl alcohol penetrated pig and human skin well with 59% and 74% of the applied radioactivity reaching the medium after 24 hours (Table 7). Moreover, almost all the parent chemical had been metabolized once it reached the medium, even after short incubation times (1 hour after topical application, data not shown). In pig skin incubations, 11 metabolites were detected in the culture medium, of which nine were identified (Figure 6, Table 7). The major peak (peak I) was either hydroxycinnamic acid or 3‐hydroxy‐3‐phenylpropanoic acid and represented 27% of the total metabolites. Cinnamic acid (peak VI) and benzoic acid (peak B) were also produced but to a lower extent (15% and 8% of the total metabolites, respectively). The remaining identified metabolites were conjugates with cysteine, glycine, glucuronide or glutathione and each represented 2%‐9% of the total metabolites (1%‐5% of the applied dose). There were eight metabolites of cinnamyl alcohol detected in human skin incubations (two of which coeluted in peak IV; Figure 6). Metabolites A and C, detected in pig skin, were below the limit of quantification for human skin incubations. The major peak (peak IV, 47% of the total metabolites) represented two metabolites, hydroxycinnamic acid and hydroxycinnamyl alcohol‐glutathione conjugates. Another major metabolite was hydroxycinnamic acid (peak I), which represented 25% of the total metabolites. From a qualitative point of view, metabolites produced by pig and human were similar but in different amounts for some metabolites.

**Table 7:** Amount of cinnamyl alcohol and its metabolites in the medium after 24 h

	% Applied dose	
	Pig	Human
Parent compound + metabolites	58.6 ± 0.1	73.6 ± 1.7
Cinnamyl alcohol (parent compound)	0.0	0.2 ± 0.03
Total metabolites	58.6 ± 0.1	73.4 ± 1.7
A (not identified)	3.9 ± 1.4	0.0*
I (HCA)	15.7 ± 1.3	18.0 ± 0.9*
II (benzoic acid glucuronide)	0.5 ± 0.04	4.6 ± 0.4*
B (benzoic acid)	4.8 ± 1.0	0.0*
C (not identified)	3.5 ± 0.9	0.0*
III (HCA‐cysteine conjugate)	5.1 ± 1.1	13.4 ± 1.2*
IV (HCA/hydroxycinnamyl alcohol‐glutathione conjugate)	10.7 ± 2.9	34.8 ± 1.4*
V (benzoic acid‐glycine conjugate)	4.4 ± 1.1	1.3 ± 0.1*
VI (cinnamic acid)	9.0 ± 0.4	0.8 ± 1.5*
VII (cinnamic acid glucuronide)	1.0 ± 0.3	0.5 ± 0.1

HCA, hydroxycinnamic acid.

Amounts of metabolites in the medium expressed as a percentage of the applied dose, mean ± SEM, four donors, *n* = 1 per donor.

* Statistical difference between pig and human skin (*P* < 0.05).

**Figure 7:**
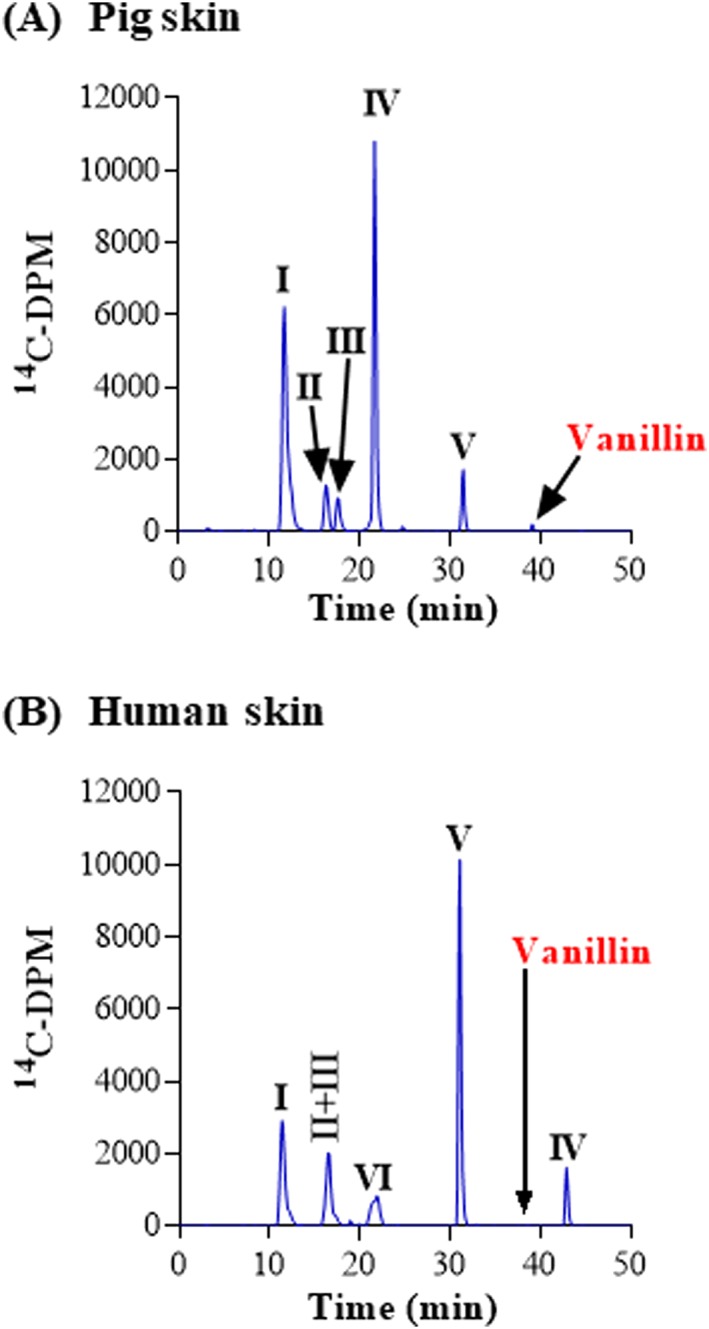
Vanillin metabolism in pig and human skin. A, Pig skin. B, Human skin. Radiochromatograms of the medium collected at 24 h from pig and human skin incubations with metabolite: I, vanillyl alcohol glucuronide + protocatechuic aldehyde glucuronide; II, vanillic acid glucuronide + vanillyl alcohol sulfate; III, vanillin glucuronide; IV, protocatechuic aldehyde; V, vanillic acid [Colour figure can be viewed at wileyonlinelibrary.com]

It is known that vanillin is converted to vanillyl alcohol as well as vanillic acid by aldehyde oxidase and to protocatechuic aldehyde by *O*‐demethylase. These metabolites are then converted to glucuronide and sulfate conjugates by UGT and SULT enzymes (Panoutsopoulos & Beedham, 2005; Strand & Scheline, 1975). In our studies, the penetration of vanillin through pig and human skin into the medium was similar, and after 24 hours, almost all the radioactivity recovered in the culture medium (~50% of the applied dose) corresponded to metabolites (Figure 7, Table 8). Six metabolites were detected and identified in the medium after application to pig skin. These were vanillyl alcohol‐glucuronide and protocatechuic aldehyde‐glucuronide (coeluting in peak I), vanillyl alcohol‐glucuronide and sulfate (coeluting with in peak II), vanillin glucuronide (peak III), protocatechuic aldehyde (peak IV) and vanillic acid (peak V) and one unidentified metabolite (peak VI) (Figure 7, Table 8). The major metabolite in pig skin was protocatechuic aldehyde (21% of the applied dose), whereas, vanillic acid was the major metabolites in human skin (34% of the applied dose). Another metabolite peak (peak I) identified to be vanillyl alcohol glucuronide and protocatechuic aldehyde glucuronide represented 21% and 10% of the applied dose in pig and humans skin, respectively. Other metabolites (vanillic acid, vanillic alcohol glucuronide and sulfate and a direct conjugate of vanillin) each represented only 3%‐6% of the applied dose in skin from both species. An additional metabolite (peak VI) was detected only in human skin incubations, which eluted after the vanillin peak but was not identified by HRMS and accounted for 1.2% of the applied dose. Vanillyl alcohol glucuronide and protocatechuic aldehyde glucuronide were also detected albeit at much lower levels (Figure 7, Table 8).

**Table 8:** Amount of vanillin and its metabolites in the medium after 24 h

	% Applied dose	
	Pig	Human
Parent compound + metabolites	54.0 ± 0.1	51.4 ± 2.3
Vanillin (parent compound)	0.1 ± 0.04	0.3 ± 0.09
Total metabolites	53.9 ± 0.1	51.1 ± 2.3
I (vannilyl alcohol‐glucuronide + protocatechuic aldehyde‐glucuronide)	21.2 ± 0.5	9.6 ± 1.1*
II (vannilyl alcohol‐glucuronide + vanillic alcohol‐sulfate)	3.3 ± 0.6	4.4 ± 1.8
III (vanillin glucuronide)	2.9 ± 0.8	
IV (Protocatechuic aldehyde)	20.7 ± 1.0	2.4 ± 1.1*
V (vanillic acid)	5.8 ± 0.1	33.5 ± 4.0*
VI (not identified)	0	1.2 ± 0.8

Amounts of metabolites in the medium expressed as a percentage of the applied dose, mean ± SEM, four donors, *n* = 1 per donor.

* Statistical difference between pig and human skin (*P* < 0.05).

## 4 DISCUSSION

The aim of these studies was to determine whether there were any species differences in the metabolism of 10 chemicals applied topically to human and pig skin explants and if pig skin can be a good alternative to human skin.

From a skin delivery point of view, whatever the compound of interest, the percentage of radioactivity (parent compound + metabolites) recovered in the culture medium after 24 hours was similar in pig and human skin. These results are in accordance with the work of Barbero and Frasch (2009) and Jacobi et al. (2007) showing that pig skin is a good surrogate for human skin in vitro permeability measurements.

Skin plays a key role in controlling not only the penetration and distribution but also the metabolism of topically applied chemicals. However, there are research gaps comparing XMEs between human and non‐human mammalian species such the pig, particularly a systematic comparison using the same culture model (albeit with small differences in the doses) within the same laboratory. As reported in the review of Oesch et al. (2014), very few data are available to conclude on the pertinence of pig skin as a surrogate of human skin regarding metabolism. The aim of this study was to therefore to compare percutaneous penetration and XME activity in the same protocol for pig and human skin.

The human and pig skin explants incubated in short‐term culture were shown able to metabolize chemicals via pathways known to involve phase I and phase II XMEs. Phase I XME‐mediated pathways included CYPs, evident as the metabolism of 7‐EC, and esterases, demonstrated by the efficient biotransformation of propyl paraben. ADH and ALDHs were also present in both pig and human skin, as cinnamyl alcohol and vanillin were both extensively and rapidly metabolized via these pathways. Phase II pathways were also shown present in skin from both species. Of the chemicals that were metabolized, all were either glucuronidated and/or sulfated. Other conjugated metabolites were also detected, such as glycine, cysteine and glutathione conjugates in incubations with cinnamyl alcohol.

Three chemicals were not metabolized by pig or human skin explants, namely, caffeine, IQ and 4‐chloroaniline. The lack of metabolism was not due to a poor bioavailability of the chemicals in the skin, as the majority of the ^14^C‐dose applied was detected in the skin and/or medium. Caffeine is metabolized by CYP1A2 in human recombinant enzymes (Ha, Chen, Krahenbuhl, & Follath, 1996) and in humans in vivo (Caubet, Elbast, Dubuc, & Brazier, 2001). It was also reported to be metabolized in liver slices, hepatocytes and human microsomes (Berthout et al., 1989) but was not metabolized in rat skin, despite extensive metabolism of many xenobiotics (Bronaugh, Stewart, & Storm, 1989). The very low abundance of CYPs in the skin, which is reported to be less than 300‐fold lower than in the liver (van Eijl et al., 2012), could be an explanation. The low abundance of CYP1A2 in the skin could also account for the lack of metabolism of IQ, which is metabolized by CYP1A2 (Shimada, Iwasaki, Martin, & Guengerich, 1989). These results are supported by Edwards et al. (1994) who showed that rate of metabolism of IQ in liver microsomes is related to the constitutive expression of CYP1A2.

The main in vivo metabolic pathways of 4‐chloroaniline include hydroxylation, *N*‐acetylation and *N‐*oxidation (IPCS, 2003). In hepatocytes, the primary metabolite, 4‐chloroacetanilide, formed by NATs was detected (Pizon et al., 2009). Human skin expresses high levels of NATs (Götz et al., 2012) and therefore we expected the *N*‐acetylated metabolite to be formed. One hypothesis as to why this metabolite was not detected in in vitro skin could be that 4‐chloroaniline was bound to the extracellular skin proteins in the epidermis, rather than entering the cells, and was therefore not available for metabolism by XMEs. This is supported by the fact that 4‐chloroaniline and its reactive metabolites bind to proteins,e.g., hemoglobin, liver and kidney proteins (IPCS, 2003) and BSA (Vandenbelt, Hansch, & Church, 1972). Likewise, in our skin explant assay, ~20% of the radioactivity detected in the culture medium was bound to proteins (most likely to BSA). Furthermore, the percentage of radioactivity in the skin was high, making extraction difficult such that additional steps of ultrasonication, stirring and a longer cryogrinding had to be included in the sample preparation process.

Species differences between pig and human were observed for some chemicals. One notable difference was the relative contribution of sulfation in pig and human skin, of which there were two examples, resorcinol and 4‐A‐3‐NP, which are metabolized to glucuronide and sulfate conjugates by UGTs and SULTs (Burnett et al., 2009; Kim & Matthews, 1987; Nystrom & Rickert, 1985). For both chemicals, the proportion of phase II metabolites is similar between the two species, but the proportion of sulfate conjugate was higher in human skin than in pig skin. This is in accordance with the known limited aryl SULT activity in pigs compared with those in humans (deBethizy & Hayes, 1989). Similar results were obtained for the metabolism of propoxur and bisphenol A in pig and human skin (van de Sandt, Rutten, & van Ommen, 1993; Zalko, Jacques, Duplan, Bruel, & Perdu, 2011).

Differences were also observed in the metabolism of cinnamyl alcohol, whereby, two unidentified metabolites were detected in pig but not human skin incubations. These differences were unlikely due to the difference in the doses used, as the production of all metabolites was linear with dose over a range of 2.4‐150 nmol/cm^2^ (data not shown). These three metabolites represented each less than 5% of the applied dose. For drug evaluation, FDA guidelines (FDA, 2017) recommended to identify and study toxicity of metabolites representing greater than 10% of the applied dose, this percentage is decreased to 5% in the Environmental Protection Agency regulatory guidance (Environmental Protection Agency (EPA), n.d.). A second species difference noted was that the major metabolite differed between the two species. Despite these differences, the metabolites formed suggested that cinnamyl alcohol was metabolized via the same metabolic pathways as those reported by others,i.e., ADH, ALDH, glutathione *S*‐transferases, glycine *N*‐acetyl transferases (Bickers et al., 2005; Cheung, Hotchkiss, & Pease, 2003).

A comparison of the metabolism of propyl paraben using pig and human skin showed that the extent of metabolism after 24 hours was close and the same metabolites were produced by both species; however, the relative amounts of each metabolite differed considerably. These results are in accordance with the work of Jewell et al. (2007) showing that pig skin has a higher ability to metabolize parabens compared to human skin; however, they concluded that pig skin was still very close to human skin and can be used as an alternative to human skin for metabolism studies. As with propyl paraben, there were species differences observed in the metabolism of vanillin. This was with respect to the major metabolite, which indicated that *O*‐demethylation is a predominant pathway in pig skin, whereas, aldehyde oxidase‐mediated metabolism is a major pathway in human skin. A second species difference was the presence of an unidentified metabolite; however, vanillin is the only chemical for which a metabolite was only formed in human and not in pig skin incubations. This could be due to differences of skin XME expression between pig and human. As this metabolite was only minor (1.2% of the applied dose) its toxicity would not be further investigated according to FDA and EMA guidelines).

## 5 CONCLUSIONS

Incorporation of skin metabolism data into the overall assessment of chemicals is key to accurate predictions of local skin toxicity and first‐pass metabolism after topical application. There are limited reports comparing percutaneous penetration and XME activities in non‐human mammalian species and human skin. The aim of this study was therefore to evaluate whether pig skin is metabolically competent and capable of generating metabolic profiles that are qualitatively and quantitatively similar to human skin, with the knowledge that such a model with exactly the same XME profile may never be found.

We demonstrated that chemicals that were not metabolized by pig skin (caffeine, IQ and 4‐chloroaniline) were also not metabolized by human skin. Likewise, all six chemicals that were metabolized by pig skin were also metabolized to a similar extent (percentage parent remaining after 24 hours) by human skin. Metabolites formed in human skin were detected in pig skin incubations, with the exception of one unidentified minor metabolite of vanillin. Three metabolites of cinnamyl alcohol were unique to pig skin but represented minor metabolites (<5% of the applied dose). There were also notable species differences in the relative amounts of common metabolites. In the case of resorcinol and 4‐A‐3‐NP, the difference in the abundance of the sulfate conjugates was in accordance with the known lack of aryl SULT activity in pigs. Therefore, pig skin is not a suitable model for use in the quantitative risk assessment of toxicity driven via a metabolite. However, our results support the use of pig skin in preliminary screening assays as an alternative to human skin to provide a qualitative indication of the potential metabolites and first‐pass metabolism of topically applied chemicals.

## CONFLICT OF INTEREST

The authors have no conflicts of interest to report.

## Supporting information


**Supporting information Table S1**. HPLC analytical conditions for metabolism measurement for each chemical. ACN = acetonitrile
**Supporting information Table S2.** Cutaneous distribution of chemicals across pig and human skin. All chemicals where tested on 4 donors of pig and human skin, except for 2‐AAF and 4 amino‐3‐nitrophenol, for which 3 donors were used. Values are mean ± SD expressed as the % of the applied dose.
