# Supplementary material for: Comparison of the metabolism of 10 chemicals in human and pig skin explants
Source: J Appl Toxicol. 2018 Oct 21;39(2):385–97. doi: 10.1002/jat.3730 (PMC6587507; doi:10.1002/jat.3730)
Supplement: Supplementary file 1 — Supporting information Table S1. HPLC analytical conditions for metabolism measurement for each chemical. ACN = acetonitrile Supporting information Table S2. Cutaneous distribution of chemicals across pig and human skin. All chemicals where tested on 4 donors of pig and human skin, except for 2‐AAF and 4 amino‐3‐nitrophenol, for which 3 donors were used. Values are mean ± SD expressed as the % of the applied dose. [file JAT-39-385-s001.doc]

**Supplementary Table 1**. HPLC analytical conditions for metabolism measurement for each chemical. ACN = acetonitrile

**Supplementary Table 2. Cutaneous distribution of chemicals across pig and human skin. All chemicals where tested on 4 donors of pig and human skin, except for 2-AAF and 4 amino-3-nitrophenol, for which 3 donors were used. Values are mean ± SD expressed as the % of the applied dose.**

| **Chemical** | **Skin surface** | | **Skin** | | **Medium** | |
| --- | --- | --- | --- | --- | --- | --- |
|  | **Pig** | **Human** | **Pig** | **Human** | **Pig** | **Human** |
| **Caffeine** | 8.8 ± 10.6 | 0.8 ± 0.8 | 8.4 ± 0.4 | 21.8 ± 2.5 | 92 ± 12.4 | 81.4 ± 3.3 |
| **4-Chloroaniline** | 0.7 ± 0.2 | 2.2 ± 0.5 | 16.3 ± 2.3 | 34.4 ± 3.8 | 82.1 ± 8.8 | 49.7 ± 4.4 |
| **IQ** | 7.8 ± 3.9 | 17.9 ± 3.7 | 33.6 ± 5.1 | 66.0 ± 12.3 | 54.8 ± 8.3 | 10.5 ± 13.4 |
| **7-EC** | 1.0 ± 0.1 | 1.4 ± 0.2 | 9.3 ± 0.4 | 24.1 ± 6.2 | 89.7 ± 3.4 | 66.7 ± 3.5 |
| **2-AAF** | 1.4 ± 0.4 | 2.8 ± 0.3 | 12.6 ± 1.5 | 40.5 ± 5.5 | 73.9 ± 1.1 | 48.3 ± 4.2 |
| **Resorcinol** | 12.3 ± 1.4 | 3.1 ± 0.8 | 9.5 ± 1.1 | 35.6 ± 4.2 | 71.0 ± 7.1 | 54.4 ± 1.5 |
| **4-Amino-3-nitrophenol** | 10.5 ± 5.1 | 3.6 ± 2.1 | 15.6 ± 5.3 | 28.6 ± 4.5 | 69.9 ± 5.8 | 62.3 ± 3.4 |
| **Cinnamyl alcohol** | 1.2 ± 1.1 | 0.2 ± 0.1 | 8.5 ± 0.8 | 16.2 ± 3.2 | 87.1 ± 2.7 | 83.5 ± 4.8 |
| **Propyl paraben** | 5.0 ± 1.8 | 2.8 ± 2.3 | 15.9 ± 3.0 | 27.6 ± 7.0 | 77.8 ± 4.8 | 61.5 ± 3.9 |
| **Vanillin** | 2.6 ± 0.2 | 2.7 ± 1.7 | 10.8 ± 3.9 | 41.7 ± 5.2 | 77.4 ± 5.1 | 60.0 ± 7.8 |
